# It Cuts Both Ways: An Annelid Model System for the Study of Regeneration in the Laboratory and in the Classroom

**DOI:** 10.3389/fcell.2021.780422

**Published:** 2021-11-29

**Authors:** Veronica G. Martinez Acosta, Fausto Arellano-Carbajal, Kathy Gillen, Kay A. Tweeten, Eduardo E. Zattara

**Affiliations:** ^1^ Department Biology, University of the Incarnate Word, San Antonio, TX, United States; ^2^ Facultad de Ciencias Naturales, Universidad Autónoma de Querétaro, Querétaro, Mexico; ^3^ Department of Biology, Kenyon College, Gambier, OH, United States; ^4^ Department of Biology, St. Catherine University, St. Paul, MN, United States; ^5^ Instituto de Investigaciones en Biodiversidad y Medio Ambiente, CONICET-Universidad Nacional del Comahue, Buenos Aires, Argentina; ^6^ Department of Invertebrate Zoology, The Smithsonian Institution, National Museum of Natural History, Washington, DC, United States; ^7^ Department of Biology, Indiana Molecular Biology Institute, Indiana University, Bloomington, IN, United States

**Keywords:** clitellate, molecular resources, stem cells, neurophysiology, invertebrate biology, STEM education

## Abstract

The mechanisms supporting regeneration and successful recovery of function have fascinated scientists and the general public for quite some time, with the earliest description of regeneration occurring in the 8th century BC through the Greek mythological story of Prometheus. While most animals demonstrate the capacity for wound-healing, the ability to initiate a developmental process that leads to a partial or complete replacement of a lost structure varies widely among animal taxa. Variation also occurs within single species based on the nature and location of the wound and the developmental stage or age of the individual. Comparative studies of cellular and molecular changes that occur both during, and following, wound healing may point to conserved genomic pathways among animals of different regenerative capacity. Such insights could revolutionize studies within the field of regenerative medicine. In this review, we focus on several closely related species of *Lumbriculus* (Clitellata: Lumbriculidae), as we present a case for revisiting the use of an annelid model system for the study of regeneration. We hope that this review will provide a primer to *Lumbriculus* biology not only for regeneration researchers but also for STEM teachers and their students.

## 1 Introduction

Regeneration—the ability to regrow body parts lost to injury—has fascinated scientists and the general public at least since the 8th century BC, as shown by the Greek myths of the Lernaean Hydra or Prometheus and his continuously regenerating liver. Although most animals demonstrate capacity for wound-healing, the ability to initiate a developmental process leading to partial or complete replacement of a lost structure varies widely among animal taxa (Bely and Nyberg, 2010). Given that humans are located towards the rather poorly-regenerating end of the spectrum, it is not surprising that we look with awe to those groups that can regrow a limb, a tail, a head, or even a complete body from a small fragment. Variability in regenerative potential is not only found between species, but may also occur within a species depending on the nature and location of the wound and the developmental stage or age of an individual. Comparative studies of cellular and molecular changes that occur both during and after wound healing may point to conserved genomic pathways among animals of different regenerative capacity. Such insight could revolutionize studies within the field of regenerative medicine.

Although the phenomenon of regeneration has been known for millennia, scientific inquiry of its developmental mechanisms began during the 18th century, and remains an active field to date. However, none of the model systems that ushered the entry of developmental biology into the molecular era (e.g., *Drosophila* fruit flies, *Mus* mice, *C. elegans* nematodes) served as good regenerative models, prompting the need for the development of alternative models to study this biologically and medically important phenomenon. Along with planarians, cnidarians, arthropods and amphibians, marine, freshwater, and terrestrial annelids have been a traditional alternative to study regeneration, and they still provide an excellent platform for this purpose. Many annelid lineages show amazing abilities to regrow an entire new body from a single fragment, while others (sometimes closely related) find themselves incapable of regenerating heads, or even tails (Zattara and Bely, 2016). Despite their foundational importance, many basic questions about the developmental mechanisms underlying annelid regeneration are still open, and only recently are being addressed using modern molecular approaches (Özpolat and Bely, 2016; Zattara, 2020; Kostyuchenko and Kozin, 2021).

One of the models that has been pivotal to annelid regeneration research is the genus *Lumbriculus* (Clitellata: Lumbriculidae). Also known as blackworms, they are taxonomically related to leeches and other mud-dwelling clitellates. Some species can regenerate an entirely new body from a fragment only 1/50th the size of the original animal. Such remarkable regenerative capabilities include the ability to recover structure and function along most of the anterior-posterior body axis. In addition, *Lumbriculus* worms subjected to long-term deprivation of nutrients will still direct resources to regeneration following amputation, further attesting to the high regenerative capacity of this annelid (Nikrad and Tweeten, 2014). Overall, studies using *Lumbriculus* offer a rich history with a focus on the morphological, cellular, physiological, and proteomic changes occurring within a regenerating worm fragment.

In this review, we first summarize the past, present, and future of regeneration research using *Lumbriculus*. To provide context for its use as a model system, we take a tour through the past—the pioneering work that started at the turn of the last century and continued during the first half of the 20th century-, the present—the overarching questions currently driving research—and the future- ushered by development of accessible sequencing and molecular techniques—of *Lumbriculus* as a study system. We then explore the potential of *Lumbriculus* outside of the research labs, as a tool for STEM Education.

## 2 The Past: The Pioneers Who Described *Lumbriculus* Regeneration

### 2.1 The Early Years: From the 18th to the Mid-20th Century

*Lumbriculus* worms have been among the earliest annelids used to experimentally investigate regeneration: working in France at the mid-18th century, Bonnet (1745) determined that a single individual could be cut in 16 pieces, and each piece would regenerate a complete worm; he also found that regenerated worms can be repeatedly cut and still retain the ability to regenerate. Over a hundred years later, the search for adult correlates of embryonic germ layers by experimental embryologists led Bülow (1883) in Germany to resume studies on this group, this time focusing on generating detailed descriptions of the regenerative process and the embryonic layer of origin of the cells that form the regenerated organs in the head and tails. This question also occupied Harriet Randolph (1892), who investigated regeneration in earthworms (Lumbricidae), sludge and water-nymph worms (Naididae), and in *Lumbriculus*. She started her work at Bryn Mawr College (PA, United States) advised by the renowned embryologist E. B. Wilson, and later at the University of Zürich (Switzerland) helped by A. Lang. Her results were published in a seminal pair of publications, in which she proposed that several mesodermal structures in the regenerate derived from segmentally iterated reserve mesodermal stem cells, which she named neoblasts, that laid dormant on the peritoneal epithelium, lateral to the ventral nerve cord (Randolph, 1891; Randolph, 1892). German, Russian and US researchers were also sectioning and studying regenerating *Lumbriculus* (von Wagner, 1900; Morgan, 1901; Iwanow, 1903; von Wagner, 1906; Morgulis, 1907; Müller, 1908; Morgulis, 1909; Krecker, 1910); research was driven by questions about the origin of the regenerated mesoderm, the differences between head and tail regeneration, and the patterns of regenerative responses that varied depending on the antero-posterior location of the regenerating tissues, the size of the fragments, and environmental and internal conditions. Most of this early phase of *Lumbriculus* research has been summarized by Stephenson (1930) in his monograph on oligochaetes.

### 2.2 Axial Regeneration: An Act in Five Stages

**FIGURE 1:**
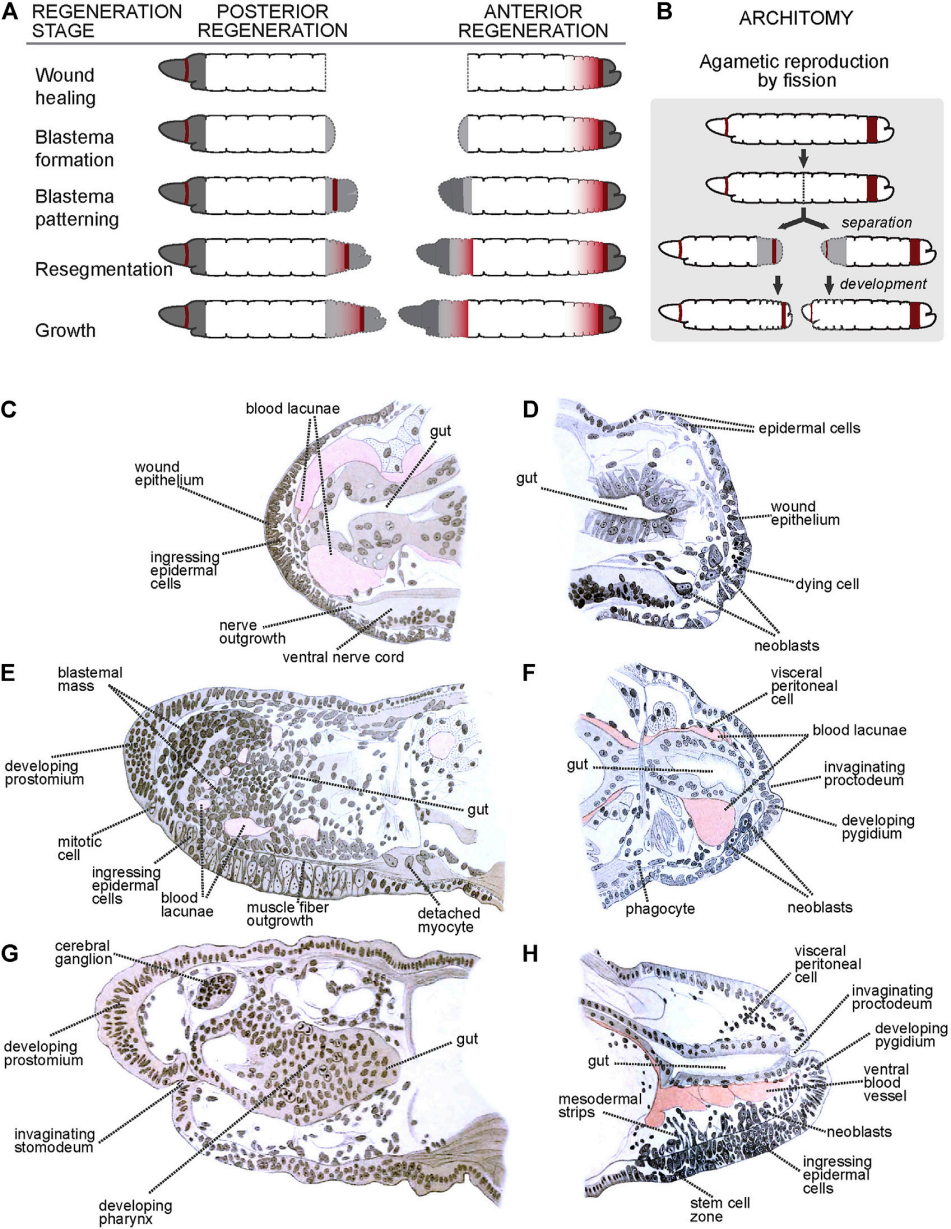
Regeneration and asexual reproduction in *Lumbriculus*. **(A)** Generic stages of annelid regeneration. Dashed line: cut/regenerated tissue; dark gray: non-segmental tissues; dark red: mitotically active areas; gray shading: differentiating segmental tissues. **(B)** Asexual reproduction by fission. Coloring as in A. **(C–H)** Histological sections through early **(C)**, middle **(E)** and late **(G)** anteriorly regenerating individuals, and early **(D)**, middle **(F)** and late **(H)** posteriorly regenerating individuals. **(C–H)** After Iwanow (1903); all labels are direct or interpreted translations of the original German labels.

Work by researchers mentioned above resulted in a very complete description of the morphological and histological processes associated with anterior (head) and posterior (tail) regeneration (Stephenson, 1930; Herlant-Meewis, 1964). After transverse amputation, the remaining worm fragments present a cut surface that can be anterior- or posterior-facing, which undergoes wound healing. After healing, anterior regeneration is triggered at anterior surfaces, resulting in the growth of a new anterior end (i.e., a head), while posterior regeneration is triggered at posterior surfaces, resulting in the growth of a new posterior end (i.e., a tail). Annelid heads and tails are organized quite differently: heads include a non-segmental terminal prostomium followed by several segmental units, an antero-dorsal cerebral ganglion, ectodermal mouth, and pharynx, and distinctively patterned ventral nerve cord ganglia; in turn, tails have a non-segmental terminal pygidium adjacent to a posterior growth zone (PGZ) which generates proximal posterior segmental units (Zattara, 2020). Thus, anterior and posterior regeneration reconstruct a considerably different suite of organs. Despite these differences, both types of regeneration processes can be divided in five stages (Figure 1A): 1) wound healing; 2) blastema formation; 3) blastema differentiation; 4) resegmentation; and 5) growth (Zattara, 2020).

#### 2.2.1 Stage 1: Wound Healing

Immediately after amputation, body wall circular muscles located adjacent to the cut site contract quickly to close off the coelomic cavity and minimize contact with the external medium. Sometimes, this fast movement closes the body wall around the cut end of the gut, which is left protruding; if this happens, the gut withdraws or pinches off the exposed end. Usually, epithelial cells from the epidermis at the edges of the cut extend to cover and seal the wound, and the same happens with the gut epithelium, which closes forming a blind end; in some cases, the edges of the epidermis and gut come into contact and fuse directly instead, closing out the wound and restoring a posterior opening (von Wagner, 1900; von Wagner, 1906). At this stage, and especially for anterior surfaces, most mitotic activity is shut down (Figures 1C,D). Damaged epithelial cells and muscle fibers degenerate and die, their remains being engulfed by phagocytes that migrate towards the wound site (Iwanow, 1903). The interstitial spaces between epidermis and gut become filled with blood lacunae.

#### 2.2.2 Stage 2: Blastema Formation

Soon after wound healing, neurites originating in nerves from the ventral nerve cord and peripheral nerves invade the wound site (Figure 1C). Around the same time, local cell proliferation is upregulated, particularly within the epidermis and gut. Many of the proliferating cells ingress from the epidermis and start forming a mass of unpigmented, undifferentiated cells known as blastema (Figure 1E). Randolph (1891), Randolph (1892), Iwanow (1903), von Wagner (1906) and Sayles (1927) describe the migration of large cells (named neoblasts) that migrate towards the wound site, proliferate there and contribute to formation of the blastema (Figures 1D,F,H); other studies in this species failed to find neoblast migration, especially during anterior regeneration (Stephenson, 1930). At this stage, it is also common to see muscle cells losing their fibrilar shapes and detaching as free myocytes into the coelomic cavity (Figure 1E).

#### 2.2.3 Stage 3: Blastema Differentiation

After accreting, the blastemal mass begins to differentiate into distal non-segmental regions: a cone-shaped prostomium in anterior regenerates (Figures 1E,G) and an anus-bearing pygidium in posterior regenerates (Figures 1F,H). In anterior regenerates, cells derived from anterodorsal epidermal proliferation and ingression begin to differentiate into a cerebral ganglion, and a band of epidermal cells located at the ventral edge of the prostomium invaginate to form a stomodeum (Figure 1G) (von Wagner, 1897; von Wagner, 1900; Iwanow, 1903). Blastemal cells around the blind end of the gut develop to form a pharynx (Figure 1G), which will eventually meet the stomodeal invagination and open as a new mouth. By this stage, neurites have already formed an anterior dorsal loop connecting the developing cerebral ganglion with the ventral nerve cord. Cells derived from ventral epidermal proliferation ingress and surround these neurites, eventually developing into the anterior ventral cord ganglia (Iwanow, 1903).

In posterior regenerates, ventral epidermal proliferation, and cell ingression, potentially along with the neoblast progeny, give rise to the primordia of the new posterior growth zone, along with the posterior ventral nerve cord ganglia (Randolph, 1892; von Wagner, 1900; Iwanow, 1903; von Wagner, 1906). At the posterior end, the epidermis invaginates towards the blind end of the gut until they connect, re-establishing the anus (von Wagner, 1906; Wenzel, 1923).

In both anterior and posterior regenerates, proliferation located proximal to the prostomium/pygidium intercalate tissues that will form the segments. Muscle fibers from existing longitudinal bands extend over the blastema until they reach the terminal caps, while circular muscle rings form apparently *de novo* (von Wagner, 1900; Iwanow, 1903; von Wagner, 1906; Wenzel, 1923). Endothelial tissue develops around the blood lacunae and restores the main ventral and dorsal vessels (Iwanow, 1903).

#### 2.2.4 Stage 4: Resegmentation

At this stage, the blastemal mass becomes organized into more discrete clusters of dorsal, lateral and ventral cells. The dorsal and lateral clusters develop into chetal sacs that secrete locomotory chaetae (von Wagner, 1906). The ventral clusters form the nerve cord ganglia. The brain completes its differentiation, and fibers of circular muscle form fine rings between the epidermis and the longitudinal muscle. At the posterior end, the regenerate transitions to developing new segments at its new posterior growth zone, as during normal growth.

#### 2.2.5 Stage 5: Growth

Regenerated structures complete differentiation and the regenerate grows in size to adjust the proportions with the original tissues to fully restore functionality.

### 2.3 Coda: Asexual Reproduction by Fission

**FIGURE 2:**
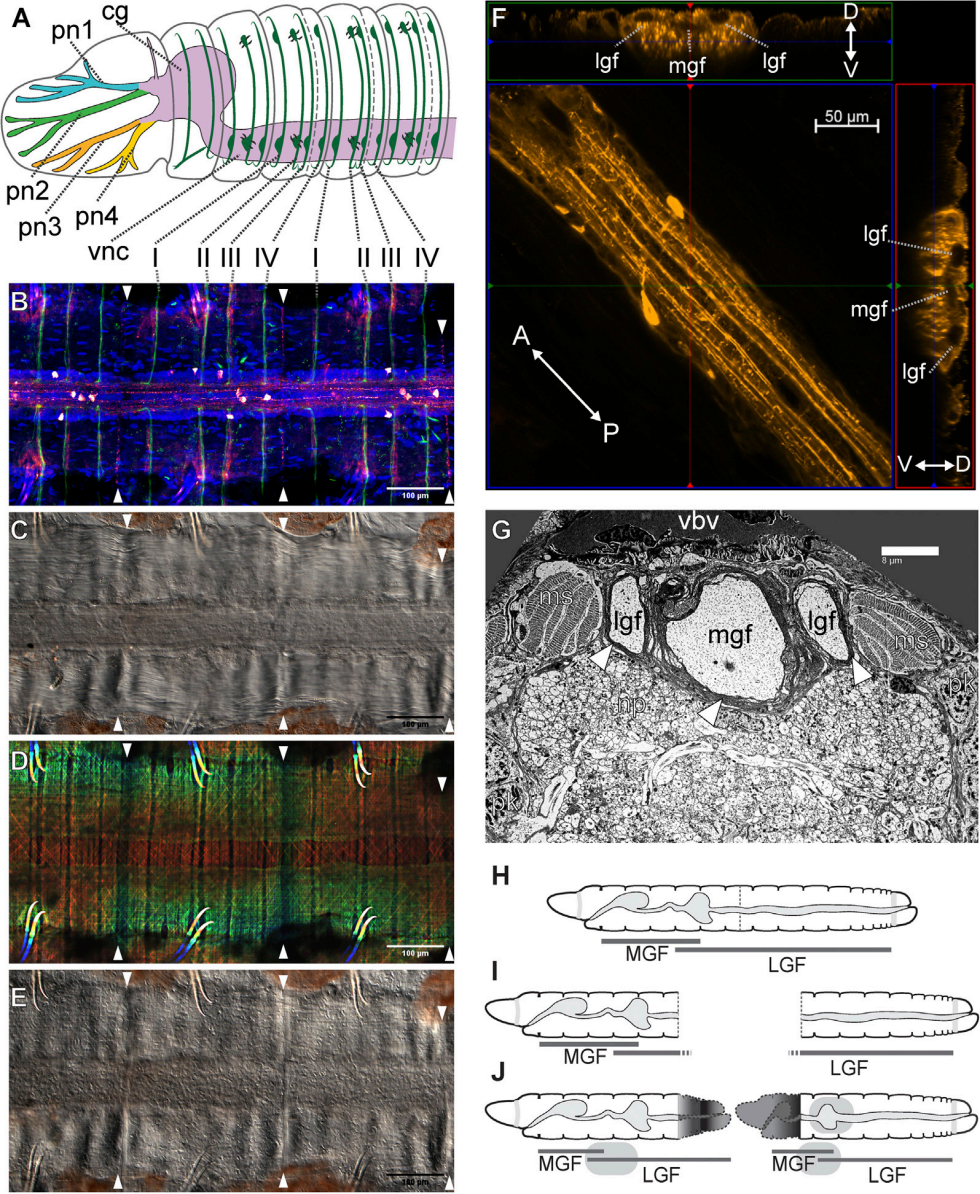
*Lumbriculus* nervous system morphology and sensory field regeneration. **(A)** Schematic representation of the anterior nervous system, showing the ventral nerve cord (vnc), dorsal cerebral ganglion (cg), prostomial nerves (pn1-4) and segmental peripheral nerves (I–IV). **(B–E)** Ventral nerve cord and peripheral nerve roots; all panels show the same whole-mounted specimen, oriented anterior to the left; arrowheads point at the segmental fission planes. **(B)** Confocal image of immunohistochemical labeling of acetylated tubulin (green) and serotonin (red-white); DNA counterstain (blue) shows cell nuclei. **(C)** Differential interference contrast (DIC) image showing the main neuropil of the nerve cord flanked by muscle bands. **(D)** Depth coded confocal stack of phalloidin-labeled F-actin showing longitudinal, circular and diagonal muscle fibers. **(E)** DIC image showing the epidermis. **(F)** Confocal stack showing a stretch of nerve cord immunolabeled against serotonin (center); the laterals are two virtual Z-sections showing the medial (mgf) and lateral giant nerve fibers (lgf). The double-headed arrows show the anterior (A)/posterior (P) and dorso (D)/ventral (V) orientation in the center and lateral panels respectively. **(G)** Transmission electron microscopy image of a thin transverse section in the anterior region of the nerve cord, showing the medial (mgf) and lateral giant nerve fibers (lgf) surrounded by myelin-like sheaths (arrowheads); ms: muscle bundle; np: neuropil; pk: perikaryon (neuronal cell body); vbv: ventral blood vessel. **(H–J)** Reestablishment of the anterior medial giant fiber (MGL) and posterior lateral giant fiber (LGL) sensory fields after amputation and regeneration. **(H)** Intact worm. **(I)** Amputated worm fragments. **(J)** Regenerated fragments redrawn after Isossimow (1926), color/nerve nomenclature after Zattara and Bely (2015).

As with many other annelids lineages, *Lumbriculus* have co-opted their amazing regenerative abilities to evolve facultative asexual reproduction (Zattara, 2012; Zattara and Bely, 2016). *Lumbriculus* are known to reproduce by breaking up into two or more fragments, each of which reforms the missing parts and become a fully functional individual (Figure 1B); this fissioning behaviour can occur within the water or inside desiccation-resistant cysts (Stephenson, 1922; Cook, 1969). Unlike injury-driven regeneration, *Lumbriculus* fragmentation results from an autotomy reflex that causes a sudden contraction of circular muscles at a very specific location along a segmental unit (Lesiuk and Drewes, 1999); in other words, and similar to other animals presenting autotomy reflexes, *Lumbriculus* have a particular “breaking plane.” This breaking plane is characterized by the presence of an epidermal serotonin immunoreactive nerve ring (Figures 2B–E, white arrowheads) (Martinez, 2005; Zattara, 2012); since treatment with nicotine, a cholinergic agonist, blocks the autotomy reflex (Lesiuk and Drewes, 1999), it is possible that the mechanism to trigger this reflex depends on acetylcholine-mediated activation of serotonergic neurons.

## 3 The Present: Burning Topics in *Lumbriculus* Regeneration Research

### 3.1 Cryptic Diversity Within *Lumbriculus*: Opening the can of Worms

For more than a century, Old and New World regeneration biologists reported working on the same species, *Lumbriculus variegatus* (Müller, 1774). In 1895, Smith described worms collected near Havana, Illinois (United States) as a separate species, *Lumbriculus inconstans*, which was later folded as a subspecies of *L. variegatus* (Brinkhurst and Cook, 1966). A further revision (Brinkhurst, 1986) describes four species: *L. variegatus* (Müller, 1774), *L. inconstans* (Smith, 1905), *L. ambiguus* (Holmquist, 1976), and *L. genitosetosus* (Holmquist, 1976). In all of these cases, the primary classification descriptors were the number and arrangement of reproductive structures in sexually mature worms. Christensen (1980) reported that worms collected in Denmark differed in their DNA content, ranging from diploid (34 chromosomes) to 11-ploid. While there are no significant morphological differences between worms from different sources, recent molecular phylogenetic analyses of various populations based on *cytochrome c oxidase subunit 1* and *16S rRNA* sequence data have shown that populations of *Lumbriculus* form differentiated genetic clusters, strongly suggesting a significant cryptic diversity of species among the initially monotypic genus (Gustafsson et al., 2009). The study found that all sequenced individuals clustered within two clearly distinct clades (aptly named Clade I and Clade II), with representatives of both clades in both Europe and North America. Clade I included worms obtained from the Environmental Protection Agency laboratory (Dultuh, MN), Aquatic Foods (Fresno, CA) and several natural habitats in Europe and North America. Clade II comprised worms from habitats in Sweden, and populations isolated from natural habitats in the United States including worms collected from the Gull Point slough in Iowa by the Drewes lab (1996). Genetic differences of up to 17.7% between clades I and II suggest that divergence in these populations occurred in the distant past. Cytological analysis (Gustafsson et al., 2009) and flow cytometry analysis of DNA content (Tweeten and Morris, 2016) of several of these genetically analyzed populations showed that worms in Clade I are polyploid while Clade II worms are diploid. DNA analysis, together with differences observed in total protein profiles and hemoglobin linker proteins (Tweeten and Morris, 2016), support designating the diploid and polyploid populations of *Lumbriculus* as distinct species.

This results in a taxonomic dilemma, as the ploidy of the type species described by Müller (1774) is not known. Research that can be inferred to have used either polyploid worms (Phipps et al., 1993) or diploid worms (Drewes and Brinkhurst, 1990) both name the worms as *Lumbriculus variegatus*. With criteria focusing on reproductive structures, the current classification system excludes many polyploid populations that, due to high chromosome numbers, likely reproduce asexually and lack reproductive structures (Christensen, 1984). Others may reproduce through parthenogenesis where reproductive structures are abnormal or substantially reduced. Clearly criteria beyond reproductive structures need to be applied in resolving the identity and diversity of species within the *Lumbriculus* complex. Recognizing the unresolved issues regarding the taxonomy of *Lumbriculus*, current taxonomic keys (Brinkhurst and Gelder, 1991) provide a set of characteristics that identify *Lumbriculus* from different sources only to the genus level: prostomium without proboscis, bifid chaetae with reduced upper tooth, lengths of 50–100 mm, anterior greenish pigmentation, and extensively branched lateral blood vessels. The worms are further described as reproducing either asexually or sexually (lack retractable penises).

The occurrence of both diploid and polyploid populations imposes a taxonomic challenge, but also provides unique opportunities for investigations of regeneration within these contexts. Since ploidy levels impact physiology, gene expression, response to environmental stresses, and rates of cell proliferation, comparative studies could be conducted, examining the impact of chromosome numbers on wound healing and downstream events occurring during regeneration. What potential differences are there in the regeneration process between diploid and polyploid forms of the worms? Are there differences in the regenerative capacity of a diploid, sexually mature worm producing cocoons compared to that of polyploid asexually reproducing worm? What differences in responses might be observed through comparative transcriptomics between diploid and polyploid *Lumbriculus* when exposed to a variety of environmental stresses?

### 3.2 Cell Migration and Proliferation: The Quest for the Neoblasts

Ever since Randolph (1891), Randolph, (1892) described the migration and proliferation of putative reserve stem cells to form the posterior blastema of *Lumbriculus*, the role of these cells has been hotly debated. Zhinkin (1932), Zhinkin (1936), Turner (1934), and Turner (1935) found that formation of both anterior and posterior structures was blocked when amputated fragments of *Lumbriculus* were exposed to x-rays to inhibit mitosis. Non-irradiated tissues, through histological analysis, showed proliferation of ectodermal cells that were linked to regeneration of nerve ganglia and the ventral nerve cord. Other cells thought to be neoblasts appeared to migrate to the wound site where they proliferated and gave rise to blood vessels and muscle cells in the regenerating tissue. Stephan-Dubois (1956) also proposed that neoblasts migrated into blastemal tissue where they proliferated and contributed to regenerating tissues. More recent experiments in which fragments of *Lumbriculus* were treated with colchicine and vinblastine, inhibitors of cell proliferation, prevented regeneration of heads and tails (Tweeten and Anderson, 2008). These results suggested that cell proliferation occurred throughout the regenerative process. Fragments allowed to regenerate for 24, 48, 72, or 120 h before being exposed to colchicine showed no further regeneration when treated with this drug. Direct evidence for cell proliferation was observed through uptake of 5-bromo-2-deoxyuridine (BrdU), a thymidine analog, into regenerating tissues (Tweeten and Anderson, 2008; Zattara and Özpolat, 2021). BrdU uptake was detected within the first 24 h of regeneration, with the greatest uptake occurring at about 120 h into regeneration.

Cell migration also was found to be essential to the regenerative process (Tweeten and Anderson, 2008). Treatment of worm fragments with locostatin and latrunculin B, inhibitors of cell migration, completely inhibited tail regeneration and partially blocked head regeneration. Other insights regarding cell migration during regeneration in *Lumbriculus* came from studies on serine proteases (Tweeten and Reiner, 2012). Given that some serine proteases play a role in the remodeling of the extracellular matrix that accompanies cell migration (Friedl and Gilmour, 2009), a fluorescently labeled reagent (Williams and Mann, 1993) that irreversibly binds to serine proteases showed high levels of these enzymes in the intestine of *Lumbriculus*. After treating worms with this reagent and then cutting the worms at the midgut level, movement of labeled intestinal cells into the developing blastema was observed. These results suggested that migration of differentiated intestinal tissue accounts, in part, for formation of the pharynx during regeneration. That serine proteases might play a role in the migration process was indicated by inhibition of head and tail regeneration by aminoethyl benzenesulfonyl fluoride, a serine protease inhibitor (Tweeten and Anderson, 2008).

Despite a long history of study, definitive evidence of neoblast migration is still scarce: wound-directed migration of neoblast-like cells has only recently been directly observed using time-lapse imaging in the freshwater clitellate *Pristina leidyi* (Zattara et al., 2016). However, 130 years after Randolph’s first paper, the role played by these migrating neoblasts in *Lumbriculus* (and other clitellates) is still unclear.

### 3.3 Regeneration and the Nervous System: Regeneration Meets Neurophysiology

The oligochaete central nervous system (CNS) generally consists of a cerebral ganglion (brain; a fused supra-esophageal ganglion) which is located in prostomium and is connected to the subesophageal ganglion and subsequently a ventral nerve cord (VNC) via two circumesophageal connectives (Stephenson, 1930; Bullock, 1965; Jamieson, 1981). In lumbriculid worms, the VNC extends down the length of the worm and gives rise to four pairs of segmental nerves within each segment (except segments 1 and 2; Figures 2A,B) (Bullock, 1965; Hessling and Westheide, 1999). These segmental nerves extend laterally around the body wall and are the source of synaptic input (sensory) and output (motor) within the clitellate CNS (Stephenson, 1930; Bullock, 1965; Jamieson, 1981). Groups of different types of neurons (sensory, motor, and interneurons) converge and are organized within each segment of the VNC (Jamieson, 1981). Axons of some of these sensory and motor neurons extend through the segmental nerves, while others extend into the neuropil of the VNC. Thus, the neuropil is a site of integration of many synaptic events that underlie the function of the worm’s neuronal circuits controlling behavioral reflexes (Bullock, 1965; Günther and Walther, 1971; Jamieson, 1981; Purschke, 2015).

*Lumbriculus* exhibits anterior-posterior gradients in behavior that are easily monitored (Drewes and Fourtner, 1990; Lesiuk and Drewes, 2001). With its tail extended into the water column, *Lumbriculus* is exposed to predation and thus has evolved rapid escape reflex behaviors that aid in survival tactics (Drewes, 1984; Zoran and Drewes, 1987). Specifically, stimulation of segments in the posterior 2/3 region of the worm’s body (Figure 2H, LGF) results in posterior shortening or tail withdrawal (Drewes, 1984; Zoran and Drewes, 1987; Drewes and Fourtner, 1989; Drewes and Fourtner, 1990). Also, touch-stimuli applied to segments found in the anterior 1/3 region of the worm’s body (Figure 2H, MGF) result in a quick anterior shortening or head withdrawal (Drewes, 1984; Zoran and Drewes, 1987; Drewes and Fourtner, 1990). Stimulation of anterior segments also results in a 180° turn or reversal locomotor response away from the aversive stimulus, whereas stimulation of posterior segments elicits rapid undulating swim movements (Drewes, 1999). These behaviors, which are specifically activated by anterior- or posterior-specific sensory inputs, are also mediated by body region-specific motor networks.

A conserved feature of virtually all oligochaetes is the presence of three giant fibers (Figures 2F,G), located in dorsal regions of the ventral nerve cord (Bullock, 1965; Jamieson, 1981; Zoran and Drewes, 1987; Hessling and Westheide, 1999; Purschke, 2015). Each of these giant nerve fibers is derived from a chain of giant axons which arise from segmentally arranged interneurons whose cell bodies are found just ventrally within the neuropil (Bullock, 1965; Günther and Walther, 1971, Jamieson, 1981: Purschke, 2015). These three giant fibers include one medial (MGF) and a pair of lateral giant (LGF) axons (Figures 2F,G). Giant axon dye-filling in *Lumbriculus* demonstrates that these axons are septate in nature; having distinct, segmental divisions separated by a membranous septum (Lybrand et al., 2020), as opposed to being syncytial, where there are no cellular divisions and thus a continuous cytoplasm between cells. Moreover, each segmentally arranged giant axon is connected via gap junctions (i.e., electrically coupled) allowing for uninterrupted through-conduction of nerve impulses along the length of the giant fiber system (Mulloney, 1970; Brink and Ramanan, 1985). Each giant fiber (GF) has 2-4 ventrally projecting collaterals and one cell body per segment. Additionally, in most oligochaetes, one lateral giant fiber (LGF) collateral forms a cross-bridge with the contralateral LGF within each segment. These interconnections are undoubtedly the basis for observed electrotonic coupling between the LGFs and the resultant bilateral synchronization of LGF action potentials during spike propagation (Drewes, 1984). It has also been demonstrated that lumbriculid giant fiber axons are ensheathed by glial cell membranes, resulting in layers of myelin surrounding the axons (Figure 2G) (Günther, 1976; Roots and Lane, 1983; Purschke, 2015; Knowles, 2017; Lybrand et al., 2020). The presence of myelination on giant fiber axons functions to increase conduction velocity along the length of the giant fibers and thus is thought to be the basis of observed rapid escape reflexes (Zoran et al., 1988; Drewes and Fourtner, 1990; Martinez et al., 2008).

Rapid escape reflexes initiated following noxious stimulus (i.e., a potential predatory threat) are mediated by the giant fiber pathways. Activation of these giant fibers via sensory stimuli (e.g. tactile or photic) results in the rapid conduction of nerve impulses down the length of the fiber that, in turn, activate motor neurons, which impinge upon longitudinal muscles responsible for body shortening (Drewes, 1984; Drewes and Fourtner, 1989; Drewes and Brinkhurst, 1990). Moreover, these rapid escape reflexes are differentially regulated by the medial and lateral giant fibers. That is, head withdrawal reflexes, in response to sensory stimuli to the anterior 1/3 of the body, are governed by the medial giant fiber (MGF) and tail reflex responses are governed by the lateral giant fibers (LGF) (Drewes and Fourtner, 1990; Lesiuk and Drewes, 2001). Interestingly, there are a few segments (Figure 2H, segments 38–58 in a worm of 150 segments) in which both a head and tail withdrawal can be elicited and both MGF and LGF activation is detected (Drewes and Fourtner, 1990). Thus, giant fiber function is governed by discrete sensory fields, with the anterior 1/3 body region falling within the MGF sensory field and the posterior 2/3 body region comprising the LGF sensory field. Interestingly, although these three giant fibers are conserved among virtually all oligochaetes, there is a fundamental difference in these rapid escape pathways between terrestrial worms (most susceptible to anterior predatory attack) and aquatic worms with tails extended from the substrate burrows (susceptible to posterior attack). Specifically, LGF sensory fields, giant fiber diameters, conduction velocities, and synaptic efficacies have become highly adapted for speed during aquatic worm (tubificid and lumbriculid) evolution (Zoran and Drewes, 1987).

The nervous system is known to play a prominent role in animal regenerative processes (Kumar and Brockes, 2012). In annelids, removing the ventral nerve cord from the segments adjacent to an amputation site can inhibit or greatly delay the regeneration process, while transplantation or deviation of the nerve cord into a wound can induce ectopic regenerates (Hyman, 1940; Herlant-Meewis, 1964; Boilly et al., 2017). This role of the nerve cord is conserved in *Lumbriculus*: regeneration occurs only in the presence of a cut end of the VNC, and the blastema begins to form next to the VNC end; furthermore, extirpation of fragments of the VNC results in the formation of ectopic lateral regenerates, adopting anterior (head) or posterior (tail) morphologies depending on the facing of the cut VNC end (von Haffner, 1928; von Haffner, 1931; Zhinkin, 1935). In turn, cell proliferation activity and neoblast migration has been proposed to be necessary for nervous system regeneration (Zhinkin, 1936). Within the nervous system, the recovery of function upon regeneration appears especially evident. Studies first carried out by electrophysiologists in the late 1970s (Günther, 1976; Drewes et al., 1978), demonstrated remarkable recovery of nervous system function. More recent studies demonstrated re-emergence of neuronal activity as early as 24-h post-amputation (Lybrand and Zoran, 2012; Lybrand et al., 2020).

The importance of nerve injury for the induction of the regenerative process has been clearly demonstrated utilizing a unique developmental paradigm which involves the formation of an ectopic head along the anterior-posterior axis of the worm (Martinez et al., 2008). Injury to the ventral nerve cord is necessary for the regeneration of proper function along the anterior-posterior axis (Martinez et al., 2008). This rapid recovery of function in the regenerating worm fragment highlights the extensive capacity for regeneration and recovery demonstrated by lumbriculid worms. Most recently, patch clamp recordings carried out with regenerating worm fragments, removed from the posterior end of the worm, demonstrated the emergence of medial giant fiber (MGF) post synaptic potentials 24 h post-amputation (Lybrand et al., 2020). These posterior regenerating fragments undergo the most drastic change in axial position, as they become more anteriorly located following the regeneration of a 7–8 segment head (Martinez et al., 2005; Martinez et al., 2006). These posterior body fragments become transformed anatomically and physiologically to match their new positional identity along the animal’s body axis (Drewes and Fourtner, 1990; Martinez et al., 2006). Specifically, these posterior body fragments exhibit transformations in touch sensory fields, giant fiber conduction velocity, axonal diameter, and other physiological properties appropriate for the fragment’s new positional identity (Drewes and Fourtner, 1990; Martinez et al., 2006). These dramatic changes within the original body fragments have been defined as morphallaxis (Morgan, 1901; Berrill, 1952; Martinez Acosta and Zoran, 2015; Kostyuchenko and Kozin, 2020; Kostyuchenko and Kozin, 2021). Morphallaxis is a pattern of regeneration where existing tissues are transformed without the involvement of stem cell differentiation (Holstein et al., 2003; Agata et al., 2007; Martinez Acosta and Zoran, 2015; Özpolat and Bely, 2016). Morphallaxis is a regenerative mechanism utilized by multiple annelids, including Enchytraeidae (Takeo et al., 2008), Syllidae (Ribeiro et al., 2018), and *Pristina* (Zattara and Bely, 2011; Özpolat et al., 2016). Morphallactic regeneration in *Lumbriculus* is especially evident within the nervous system (Martinez et al., 2005; Zoran and Martinez, 2009; Martinez Acosta and Zoran, 2015), where non-invasive extracellular electrophysiology demonstrates a rapid switching between Medial Giant Fiber (MGF) to Lateral Giant Fiber (LGF) pathways in the transforming posterior segments. In less than 24 h post amputation, these posterior-most fragments display MGF activity (Figures 2I,J) (Lybrand and Zoran, 2012). The speed with which the MGF pathway becomes functionally activated in these posterior regenerating fragments demonstrates the remarkable plasticity of the nervous system in *Lumbriculus*, which is poised for regeneration and recovery of function. Continued work will help elucidate the exact physiological repertoire used for this incredible plasticity event.

## 4 The Future: *Lumbriculus* Enters the Genomics Era

Sydney Brenner (2002) said “Progress in science depends on new techniques, new discoveries and new ideas, probably in that order.” *Lumbriculus* research has taken an important step into the genomics era with a recent transcriptomic study comparing the profiles of regenerating and non-regenerating worms (Tellez-Garcia et al., 2021). This work identified 136 transcripts likely to be differentially expressed during early regeneration, 73 of which were potentially protein-coding and had significant BLASTp hits to known proteins; among them were *bmi1b*, *Hsp60*, *vdr*, *BHMT*, *paics*, *Gls2* and several *vwdes*—all genes found to be also differentially expressed during regeneration of annelids or other systems. Besides highlighting some interesting candidate genes, this study generated a fundamental resource by providing a comprehensive database of sequences from genes expressed during *Lumbriculus* regeneration.

Additional sequence data is available from transcriptomic and phylogenomic studies, including RNAseq data for specimens from Sweden (SRX2649483) (Anderson et al., 2017), and genomic DNA short read sequences from Denmark (SRX9009164) and Sweden (SRX5630329) (Phillips et al., 2019). With an estimated genome size of 2.64 Gbp (Tweeten and Morris, 2016), which is larger than that of the domestic mouse, sequencing and assembling a reasonable quality genome draft is not a trivial task, especially given the relatively small size of the currently active *Lumbriculus* research community. Even so, the existing transcriptomic resources currently available are already pushing research forward, as specific genes and developmental pathways begin to be investigated.

Generation and sharing of molecular resources among researchers are important steps in moving *Lumbriculus* research into the modern molecular era. Gene expression analyses are powerful tools for screening of genes that may be involved in regeneration. Thus, the development of techniques for gene expression analysis is of utmost importance. A step toward this work is the optimization of real-time PCR protocols by the Martinez Acosta and Gillen labs which will reliably quantify expression of genes of interest (Quesada et al., 2015; LaRocca-Stravalle et al., 2020; Fischer et al., 2021).

Culturing of *Lumbriculus* poses limitations for this genetic work, due to the lack of sexual reproduction in the laboratory. *Lumbriculus* is collected in the field as sexually reproducing populations during summer months (Tweeten and Morris, 2016). The Drewes and Tweeten Labs have successfully raised cocoons in the lab which were collected in the field, showing promise for studies of regeneration during different developmental stages and for general investigations underlying genetic mechanisms in this remarkable worm (Drewes and Brinkhurst, 1990; Tweeten and Vang, 2011; Tweeten and Abitz, 2012; Tweeten and Morris, 2016). Access to sexually reproducing individuals has also opened up new avenues of research on questions related to sexual reproduction, including seasonal variation in cocoon production, anatomical location of reproductive structures within the worm, sperm morphology and formation, degradation of reproductive structures under laboratory conditions, and regulation of sexual reproduction in these worms. Transcriptomes from sexually reproducing populations of *Lumbriculus* and from asexually reproducing populations are being generated and studies comparing these transcriptomes are underway. Some questions of interest include: What are the properties of the DNA-binding proteins that package DNA into the sperm of *Lumbriculus*? What type of mucin proteins are produced by sexually reproducing worms and released into cocoons to cushion embryos during their development in the environment? What is the composition of the yolk proteins present in the eggs produced by sexually reproducing *Lumbriculus*? Are genes for DM proteins (ie., *Dmrt*), which regulate sexual development, differentially expressed in tissues from sexually reproducing worms? How similar or different are they to DM proteins from other animals? Characterization of these proteins would provide insights into mechanisms leading to sexual versus asexual modes of reproduction in *Lumbriculus*.

Further development of genomic methods will move *Lumbriculus* research beyond correlation and shift the focus of future work toward demonstrating the functional significance of gene expression changes. In particular, successful application of reverse genetic techniques such as RNA interference (RNAi) and the CRISPR-Cas endonuclease system would allow assessing gene function and drastically change the playing field for *Lumbriculus* regeneration studies. Work aimed to develop these techniques is already ongoing in several labs, and this research has benefited from fluid communication, data and resource exchange, and collaborative work.

### 4.1 *Lumbriculus* as a Model for Epigenetic Regulation of Regeneration

Regeneration depends on proliferation and differentiation and requires marked changes in gene expression programs based on epigenetic modifications (Barrero and Izpisúa Belmonte, 2011; Hamada et al., 2015; Rouhana and Tasaki, 2015). Epigenetic regulation of regeneration is achieved by three main mechanisms: DNA methylation, histone modification and noncoding RNAs (Rouhana and Tasaki, 2015). There are already reports of the relevance of epigenetic regulation for development and regeneration of annelids (Giani et al., 2011; Niwa et al., 2013; Kozin and Kostyuchenko, 2015; Bhambri et al., 2018; Bicho et al., 2020; Singh Patel et al., 2020; Planques et al., 2021). An analysis of the regeneration transcriptome of *Lumbriculus variegatus* (Tellez-Garcia et al., 2021) in search for transcripts encoding for writers and erasers of DNA methylation revealed genes encoding for DNA methyltransferases and several ten-eleven translocation proteins, as well as histone acetyltransferases, histone deacetylase, histone methyltransferases, and histone demethylases. Furthermore, 44,097 potential lncRNAs were identified, of which 13 were upregulated during *Lumbriculus* regeneration. Among the differentially expressed transcripts was bmi1b (Polycomb complex protein BMI-1-B), which has been implicated in regeneration in mammals (Fukuda et al., 2012). Moreover, piwi genes were also found in the *Lumbriculus* transcriptome (Tellez-Garcia et al., 2021). Thus, despite the currently limited in terms of molecular and genetic data, a brief analysis of the epigenetic regulation repertoire in *Lumbriculus* suggests that this annelid has the potential to be developed as a new model to study epigenetic regulation during regeneration.

## 5 An Accessible Model for the Lab and the Classroom

One of the main advantages of *Lumbriculus* as a study system includes its accessibility; individuals can be collected from the field in many temperate regions or acquired from several commercial suppliers. *Lumbriculus* spp. are easy to care for with minimal equipment—they only require containers, bubblers, and food—and many populations will readily reproduce by asexual fragmentation, allowing the attainment of a large number of worms in laboratory settings. This yields an advantage not only for research, but for life sciences educators as well, as blackworms serve as a well-established tool for science education. Current use occurs across high school and college classrooms to demonstrate concepts in Cellular Biology, Physiology, Animal Behavior, Biomechanics, Development, and Invertebrate Biology, both using guided inquiry as well as more advanced independent studies.

### 5.1 Procurement of *Lumbriculus*

**FIGURE 3:**
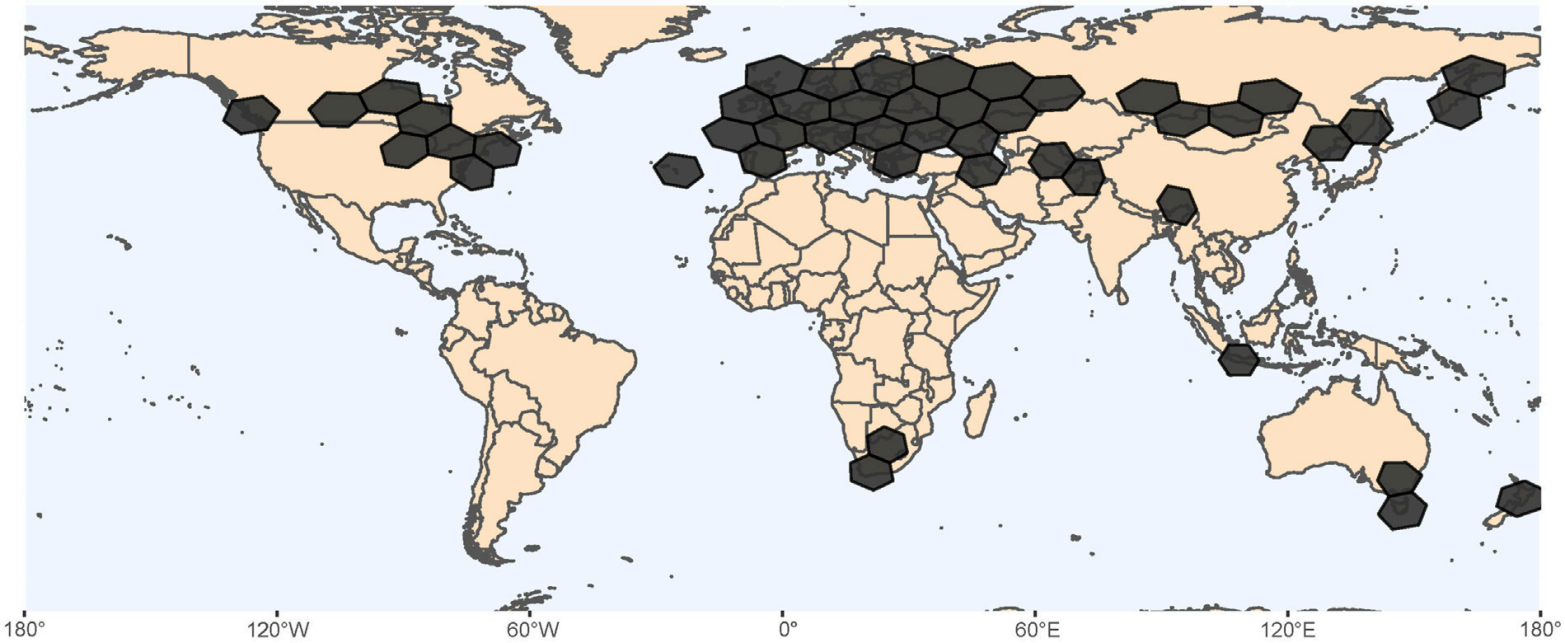
Geographic distribution of *Lumbriculus* spp, based on occurrence records found at the Global Biodiversity Information Facility (GBIF.org, 2021). Hexagons in South Africa, Indonesia, Australia and New Zealand represent 98 records that would need additional verification.

*Lumbriculus* are available from commercial suppliers such as Aquatic Foods (Fresno, CA United States), Eastern Aquatics (Lancaster, PA), and Aquarem (Mexico DF, Mexico), that sell them as blackworms or mudworms for use in aquaculture. Various biological stores sell *Lumbriculus* for educational purposes, providing supporting curriculum kits directly to K-12 biology teachers, like Carolina Biological (Burlington, NC, United States) and Flinn Scientific (Batavia, IL. United States). *Lumbriculus* can also be obtained from the Environmental Protection Agency (EPA) Laboratory in Duluth, MN, United States where a culture has been maintained since the 1980s. All of these sources of *Lumbriculus* derive from polyploid populations, with chromosome counts compatible with at least 11-ploid to 12-ploid worms (Tweeten and Morris, 2016). *Lumbriculus* can also be collected from freshwater habitats throughout Eurasia, North America, and regions of the Northern Pacific (Figure 3). Lakes and ponds with standing or slow-moving water provide potential collection sites, especially where shorelines have deciduous trees, sedges, rushes, and cattails that contribute decaying plant material that accumulates in the shallow water along the edges of the lakes (Brinkhurst and Gelder, 1991). The leaf litter, grasses, and sediments along the edges of sloughs, marshes, and drainage ditches that persistently retain water are good collection sites, due to the water being more still and shallow. Sometimes specimens can be found further from the shoreline in algal mats growing on the surface of the water. In the United States, both diploid (from Minnesota, Wisconsin, Iowa, North Dakota) and polyploid (from California and Oregon) populations of *Lumbriculus* have been collected from natural habitats (Gustafsson et al., 2009; Tweeten and Morris, 2016). Several of the diploid populations have been observed to sexually reproduce during the summer months, producing cocoons for a limited period in the laboratory following their collection from natural habitats (Drewes and Brinkhurst, 1990; Tweeten and Morris, 2016). In Europe, diploid, polyploid, and sympatric populations of *Lumbriculus* have been collected from natural habitats (Christensen, 1980; Gustafsson et al., 2009).

### 5.2 Culturing of *Lumbriculus*

Whether obtained commercially or collected from the environment, *Lumbriculus* can then be easily maintained in the laboratory in spring water, while some labs have successfully used dechlorinated tap water. Worms are fed with fish flakes or pellets such as Tetramin^®^, rolled oats, and spirulina over a range of temperatures (typically 15°C to room temperature). Microbes in the cultures also serve as a source of nutrition. Strips of brown paper towels are added to mimic the leaf litter of natural habitats. For bioaccumulation experiments, sandy or other fine sediment types can be added to the cultures (Sardo et al., 2007). Some labs aerate the worm cultures, especially if large numbers of worms are being maintained. Water quality is closely monitored in large cultures. Various populations of *Lumbriculus* collected from natural habitats do not fare well when transferred to spring water. These are best maintained in water from the collection site that is filtered to remove particulates. Reproduction under laboratory conditions is almost exclusively by architomy followed by regeneration (Drewes and Brinkhurst, 1990; Martinez et al., 2006). As the worms proliferate, they can be subcultured. Ectoparasites can sometimes be associated with *Lumbriculus* obtained commercially or from natural habitats; their levels can get to a point where survival of the worms is jeopardized and the cultures crash. However, these ectoparasites can be removed by treating cultures with 0.6% sodium chloride in spring water. The EPA lab (Duluth, MN) found that salt provokes release of ectoparasites from the surface of the worms. Overall, the general culturing of *Lumbriculus* is carried out with ease, thus providing a reliable source for experimentation.

### 5.3 *Lumbriculus* in the Classroom: The Legacy of Charlie Drewes

**FIGURE 4:**
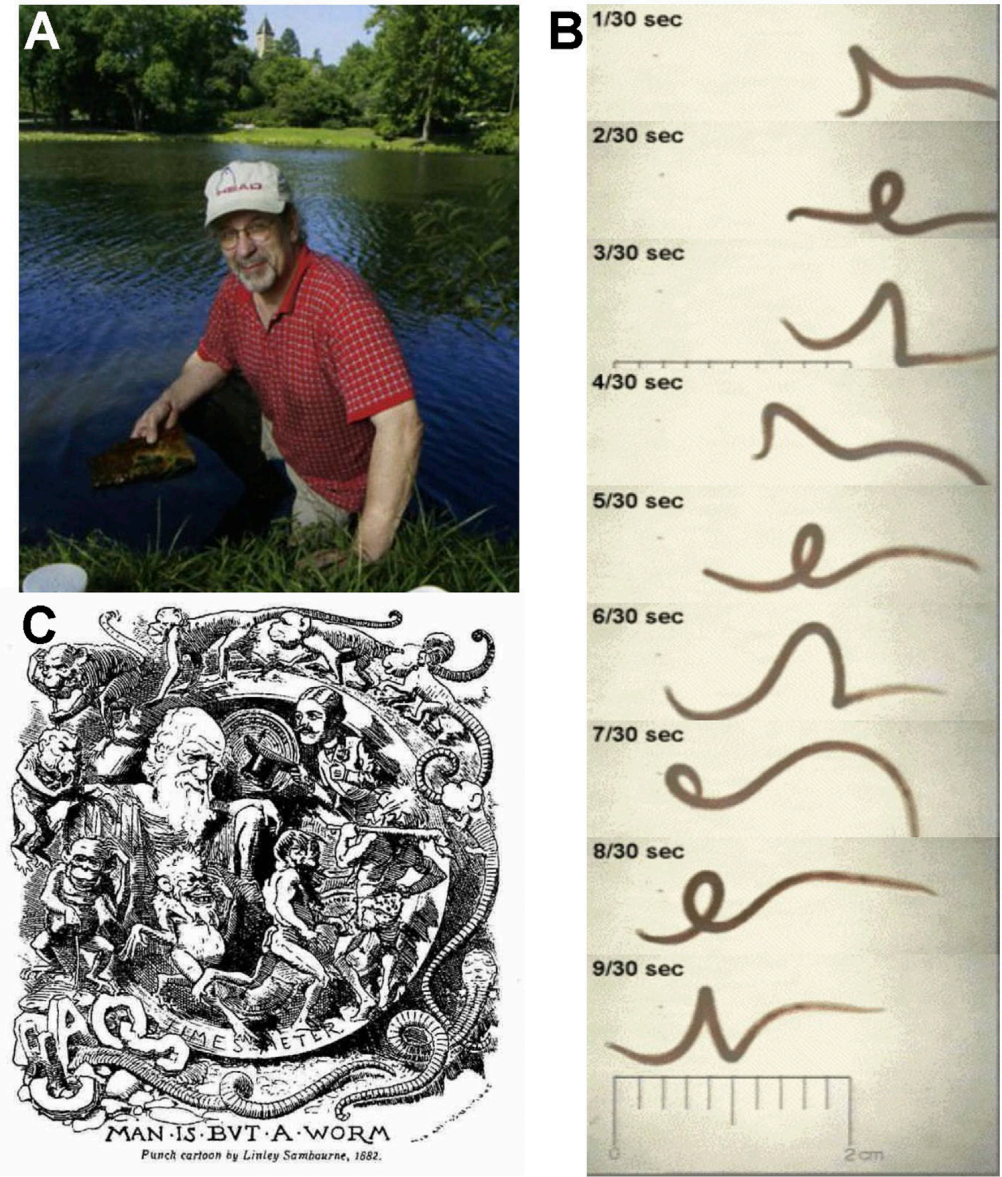
**(A)** Charlie Drewes collecting *Lumbriculus*, which he first proposed in 1996 as an inexpensive and accessible organism for high school and university student laboratory experiences. **(B)** Video frame capture of helical swimming behavior elicited when the posterior segments are stimulated, Example of online resources available for classroom use of *Lumbriculus* to study basic biology. Many of the educational outreach resources developed by Dr. Drewes incorporating invertebrates into student projects and activities are preserved and accessible at the C. Drewes website maintained by Iowa State University: http://www.eeob.iastate.edu/faculty/DrewesC/htdocs/. **(C)** *Man is but a worm*. Charlie was known to appreciate plays on words and was a master of disguising biological ideas within the puns he often shared. He enjoyed this caricature published in December 1881 following Darwin’s last publication, *The Formation of Vegetable Mould Through the Action of Worms*.

While used extensively in monitoring of environments for pollutants and toxicity testing of industrial compounds (Goodnight, 1973; Hornig, 1980; Chapman and Brinkhurst, 1984; Phipps et al., 1993), *Lumbriculus* was first proposed by Charles Drewes (1996) as an inexpensive and accessible organism for high school and university student laboratory experiences (Figure 4). Drewes’ outreach to teachers and students began with development of laboratory exercises through which students explore segmental pattern formation during regeneration in *Lumbriculus*. Through detailed supply lists, descriptions of techniques, and examples of experimental design, Drewes described how students could generate and maintain worm fragments in the teaching lab. Through observations of regenerating fragments, students then learn about morphallaxis, the developmental process of reorganization that occurs as *Lumbriculus* regenerates anterior segments. As restoration of tissues and anatomical structures such as blood vessels are monitored, anterior regeneration is compared to posterior regeneration. Students also explore the influence of amputation location along the anterior-posterior axis and fragment size on numbers of regenerated segments. Generation in the laboratory of worm fragments by amputation severs the ventral nerve cord, disrupting the locomotory responses typical of anterior and posterior regions of *Lumbriculus*. As students monitor the fragments for recovery of different locomotory functions such as swimming, crawling, reversal behaviors, the role of the nervous system in the regenerative process can be explored (Drewes and Cain, 1999). Since then, a number of other *Lumbriculus*-based activities related to regeneration, physiology, and neurobiology have been designed for teaching laboratories. These hands-on activities have been made accessible online and published in journals ranging from Tested Studies for Laboratory Teaching Proceedings of the Association for Biology Laboratory Education (Bohrer, 2006; Killian and Baker, 2013) to Science Scope (Straus and Chudler, 2015), and Bioscene (Ryan and Elwess, 2017). These lab activities provide students with opportunities to learn about the anatomy of the worm’s circulatory system, observe behaviors which are easily correlated to restoration of nervous system function during regeneration, develop observational skills, and draw on the scientific literature to inform their approach to inquiry. Through such examples and protocols highlighting the use of materials and equipment that could easily be found in a high school or college laboratory, these articles model biological inquiry as it is carried out in scientific laboratories across the world, while also introducing the student to the important roles played by annelids within the greater environment. Students are immersed in the scientific process, formulating research questions, generating predictions, and designing experiments. Within a two to 3-h lab period, students are identifying experimental variables, setting up experiments, and collecting data using *Lumbriculus*. In addition to learning about the process of science, each laboratory investigation incorporates various methods for mathematical modeling and statistical testing of data which was collected by the student, further enhancing the learning experience through the application of quantitative skills (Killian and Baker, 2013). The dissemination of hands-on *Lumbriculus* activities has inspired the development of a growing community of educators that offer creative modifications and improvements to student learning experiences (Killian and Baker, 2013; Ryan and Elwess, 2017). For example, experimentation with *Lumbriculus* in the biology classroom helps students make sense of physiological concepts and functions in vertebrates, like themselves. Experiments that would be difficult or impossible to do in more complex systems can readily be done with *Lumbriculus* (Bohrer, 2006; Straus and Chudler, 2015). While designing projects to study the effect of various chemical and environmental factors on the regenerative process in *Lumbriculus*, students can also explore why regeneration of tissues is so limited in most other animals.

### 5.4 *Lumbriculus* in Undergraduate Research Training

Because so many aspects of the physiology, behavior, cell, and molecular interactions of *Lumbriculus* remain to be characterized, this annelid is an excellent organism for course-based undergraduate research experiences. At St. Catherine University, original research projects focused on *Lumbriculus* have been incorporated into several upper-level biology courses. Students learn cellular, molecular, and immunological techniques while applying them to basic questions about the structure and physiology of this annelid. For example, students in an immunology course used immunohistochemistry to study the distribution of fibronectin, laminin and collagen in cross-sections of worm tissue while antibodies against peptidoglycan were used to compare the quantity of microbes in the intestines of fed and starved worms. In the laboratory component of a “Molecular Biology: Proteins” course, students screened a *Lumbriculus* transcriptome for actin sequences and constructed a phylogenetic tree that revealed that the actin from *Lumbriculus* was more closely related to vertebrate cytoplasmic β-actins than vertebrate cardiac, smooth, or skeletal muscle α-actins. This relationship was further supported as students conducted Western blots on worm homogenates, finding that antibodies against vertebrate β-actin reacted more strongly with *Lumbriculus* actin than antibodies against vertebrate α-actin. Incorporated into the laboratory component of courses, the projects increase the research capacity of biology programs at small institutions. Students who otherwise might not have participated in a formal research experience find themselves immersed in the scientific process. In many cases, the course projects have expanded into collaborative research studies in faculty research labs that have resulted in student presentations at scientific conferences and publications in peer-reviewed journals with students as co-authors (Crisp et al., 2010).

These examples show how *Lumbriculus* is an ideal organism through which students can explore their interest and aptitude for science. Engagement in the research process while asking original questions and contributing to the scientific knowledge base has enhanced student motivation and satisfaction with their learning (Tweeten et al., 2007). Projects have generated original results which have been published in scientific journals, like Comparative Biochemistry and Physiology (Crisp et al., 2010) and Invertebrate Biology (Tweeten and Reiner, 2012; Tweeten and Morris, 2016) as well as journals that focus on publication of research conducted by undergraduates, such as BIOS (Tweeten and Anderson, 2008) and Impulse (Halfmann and Crisp, 2011).

## 6 Concluding Remarks

Thanks to the work of past and present researchers working on *Lumbriculus* regeneration and related topics, we now have a very firm foundation to launch new forays into many unresolved questions regarding the genetic, developmental, physiological, ecological and evolutionary underpinnings of these worms’ amazing regenerative abilities. In doing so, conserved, and novel mechanisms driving regeneration might be unveiled, informing development of alternative biomedical approaches. Furthermore, the research process will help current and future researchers learn many lessons about molecular and developmental biology, physiology and ecology that will become part of their professional toolkit whether they stick with *Lumbriculus* or move on to work on other systems. In summary, and despite the challenges associated with working with a non-traditional study system as *Lumbriculus* (such as lack of a reference genome, relatively underdeveloped molecular tools, and a much smaller knowledge base relative to models like *Drosophila*, *C. elegans* or mice), we think that the advantages of this organism—ease of procurement and culture, fast and robust regenerative abilities, rich research history, considerable ecological and genetic diversity, and a large spectrum of open questions with significant biological and biomedical relevance—render it a superb organism for regeneration research, either in a science research lab or in elementary, middle and undergraduate classrooms. We hope this review article will foster further work on *Lumbriculus* regeneration in research labs and encourage expanded use of these worms in the teaching labs; in turn, the work and questions of students are bound to spark new ideas for the research lab. Two heads are better than one.

## References

[B1] Agata K. Saito Y. Nakajima E. (2007). Unifying Principles of Regeneration I: Epimorphosis versus Morphallaxis. Dev. Growth Differ. 49 (2), 73–78. 10.1111/j.1440-169X.2007.00919.x 17335428

[B2] Anderson F. E. Williams B. W. Horn K. M. Erséus C. Halanych K. M. Santos S. R. (2017). Phylogenomic Analyses of Crassiclitellata Support Major Northern and Southern Hemisphere Clades and a Pangaean Origin for Earthworms. BMC Evol. Biol. 17, 123. 10.1186/s12862-017-0973-4 28558722 PMC5450073

[B3] Barrero M. J. Izpisúa Belmonte J. C. (2011). Regenerating the Epigenome. EMBO Rep. 12, 208–215. 10.1038/embor.2011.10 21311559 PMC3059915

[B4] Bely A. E. Nyberg K. G. (2010). Evolution of Animal Regeneration: Re-emergence of a Field. Trends Ecol. Evol. 25, 161–170. 10.1016/j.tree.2009.08.005 19800144

[B5] Berrill N. J. (1952). Regeneraton and Budding in Worms. Biol. Rev. 27, 401–438. 10.1111/j.1469-185x.1952.tb01512.x

[B6] Bhambri A. Dhaunta N. Patel S. S. Hardikar M. Bhatt A. Srikakulam N. (2018). Large Scale Changes in the Transcriptome of *Eisenia fetida* during Regeneration. PLOS ONE 13, e0204234. 10.1371/journal.pone.0204234 30260966 PMC6160089

[B7] Bicho R. C. Scott-Fordsmand J. J. Amorim M. J. B. (2020). Developing an Epigenetics Model Species - from Blastula to Mature Adult, Life Cycle Methylation Profile of *Enchytraeus crypticus* (Oligochaete). Sci. Total Environ. 732, 139079. 10.1016/j.scitotenv.2020.139079 32428769

[B8] Bohrer K. E. (2006). Effects of Drugs on Pulsation Rate of *Lumbriculus variegatus* (Blackworms). Assoc. Biol. Lab. Edu. 27, 127–146.

[B9] Boilly B. Boilly-Marer Y. Bely A. E. (2017). Regulation of Dorso-Ventral Polarity by the Nerve Cord during Annelid Regeneration: A Review of Experimental Evidence. Regeneration 4, 54–68. 10.1002/reg2.78 28616245 PMC5469730

[B10] Bonnet C. (1745). Traité d’insectologie . Seconde partie. Observations sur quelques espèces de vers d’eau douce, qui coupés par morceaux, deviennent autant d’animaux complets. A Paris, Chez Durand. Available at: http://archive.org/details/traitdinsectolog02bonn (Accessed February 6, 2016).

[B11] Brenner S. (2002). Life Sentences: Detective Rummage Investigates. Genome Biol. 3, comment1013.1–comment1013.2. 10.1186/gb-2002-3-2-comment1003

[B12] Brink P. R. Ramanan S. V. (1985). A Model for the Diffusion of Fluorescent Probes in the Septate Giant Axon of Earthworm. Axoplasmic Diffusion and Junctional Membrane Permeability. Biophys. J. 48, 299–309. 10.1016/S0006-3495(85)83783-8 4052564 PMC1329321

[B13] Brinkhurst R. O. Cook D. G. (1966). Studies on the North American Aquatic Oligochaeta III: Lumbriculidae and Additional Notes and Records of Other Families. Proc. Acad. Nat. Sci. Philadelphia 118, 1–33.

[B14] Brinkhurst R. O. Gelder S. (1991). “Annelida: Oligochaeta and Branchiobdellida,” in Ecology and Classification of North American Freshwater Invertebrates (New York, NY: Academic Press), 431–463.

[B15] Brinkhurst R. O. (1986). Guide to the Freshwater Aquatic Microdrile Oligochaetes of North America. Can. Spec. Publ. Fish. Aquat. Sci. 84, 259.

[B16] Bullock T. H. (1965). “Annelida,” in Structure and Function in the Nervous System of Invertebrates. Editors Bullock T. H. Horridge G. A. (San Francisco: Freeman), 661–789.

[B17] Bülow C. (1883). Die Keimscichten des wachsenden Schwanzendes von *Lumbriculus variegatus* nebst Beiträgen zur Anatomie und Histologie dieses Wurmes. Z. für wissenschaftliche Zoologie 39, 64–96.

[B18] Chapman P. M. Brinkhurst R. O. (1984). Lethal and Sublethal Tolerances of Aquatic Oligochaetes with Reference to Their Use as a Biotic index of Pollution. Hydrobiologia 115, 139–144. 10.1007/BF00027908

[B19] Christensen B. (1980). *Annelida*. Berlin-Stutgart: Gebrüder Borntraeger. Available at: https://www.schweizerbart.de/publications/detail/isbn/9783443260101 (Accessed September 13, 2021).

[B20] Christensen B. (1984). Asexual Propagation and Reproductive Strategies in Aquatic Oligochaeta. Hydrobiologia 115, 91–95. 10.1007/bf00027898

[B21] Cook D. G. (1969). Observations on the Life History and Ecology of Some Lumbriculidae (Annelida, Oligochaeta). Hydrobiologia 34, 561–574. 10.1007/BF00045410

[B22] Crisp K. M. Grupe R. E. Lobsang T. T. Yang X. (2010). Biogenic Amines Modulate Pulse Rate in the Dorsal Blood Vessel of *Lumbriculus variegatus* . Comp. Biochem. Physiol. C: Toxicol. Pharmacol. 151, 467–472. 10.1016/j.cbpc.2010.02.003 20167287

[B23] Drewes C. Cain K. (1999). As the Worm Turns: Locomotion in a Freshwater Oligochaete Worm. Am. Biol. Teach. 61, 438–442. 10.2307/4450725

[B24] Drewes C. D. Brinkhurst R. O. (1990). Giant Nerve Fibers and Rapid Escape Reflexes in Newly Hatched Aquatic oligochaetes, *Lumbriculus variegatus* (Family Lumbriculidae). Invertebrate Reprod. Develop. 17, 91–95. 10.1080/07924259.1990.9672095

[B25] Drewes C. D. (1984). “Escape Reflexes in Earthworms and Other Annelids,” in Neural Mechanisms of Startle Behavior. Editor Eaton R. C. (Boston, MA: Springer US), 43–91. 10.1007/978-1-4899-2286-1_3

[B26] Drewes C. D. Fourtner C. R. (1989). Hindsight and Rapid Escape in a Freshwater Oligochaete. Biol. Bull. 177, 363–371. 10.2307/1541596 29300597

[B27] Drewes C. D. Fourtner C. R. (1990). Morphallaxis in an Aquatic Oligochaete, *Lumbriculus variegatus*: Reorganization of Escape Reflexes in Regenerating Body Fragments. Develop. Biol. 138, 94–103. 10.1016/0012-1606(90)90179-M 2307291

[B28] Drewes C. D. (1996). “Heads or Tails? Patterns of Segmental Regeneration in a Freshwater Oligochaete,” in Tested studies for laboratory teaching Proceedings of the 17th Workshop/Conference of the Association for Biology Laboratory Education (ABLE), West Lafayette, IN, June 6–10, 1995. Editor Glase J. C. (Purdue University), 23–35. Available at: https://www.ableweb.org/biologylabs/wp-content/uploads/volumes/vol-17/2-drewes.pdf .

[B29] Drewes C. D. (1999). “Helical Swimming and Body Reversal Behaviors in *Lumbriculus variegatus* (Annelida: Clitellata: Lumbriculidae),” in Aquatic Oligochaetes Developments in Hydrobiology. Editors Healy B. M. Reynoldson T. B. Coates K. A. (Dordrecht: Springer Netherlands), 263–269. 10.1007/978-94-011-4207-6_26

[B30] Drewes C. D. Landa K. B. McFall J. L. (1978). Giant Nerve Fibre Activity in Intact, Freely Moving Earthworms. J. Exp. Biol. 72, 217–227. 10.1242/jeb.72.1.217 624897

[B31] Fischer F. LaRocca-Stravalle Z. Gillen K. (2021). Regeneration in *Lumbriculus variegatus* Entails Differential Expression of Telomerase Reverse Transcriptase. Integr. Comp. Biol. 61, e1113–1114.

[B32] Friedl P. Gilmour D. (2009). Collective Cell Migration in Morphogenesis, Regeneration and Cancer. Nat. Rev. Mol. Cel Biol. 10, 445–457. 10.1038/nrm2720 19546857

[B33] Fukuda A. Morris J. P. Hebrok M. (2012). Bmi1 Is Required for Regeneration of the Exocrine Pancreas in Mice. Gastroenterology 143, 821–831. 10.1053/j.gastro.2012.05.009 22609312 PMC3485080

[B34] GBIF.org (2021). *Lumbriculus* Occurrences Structured Query. Available at: https://www.gbif.org/occurrence/download/0001666-210914110416597 (Accessed September 15, 2021).

[B35] Giani V. C. Jr. Yamaguchi E. Boyle M. J. Seaver E. C. (2011). Somatic and Germline Expression of Piwi during Development and Regeneration in the marine Polychaete Annelid *Capitella teleta* . Evodevo 2, 10. 10.1186/2041-9139-2-10 21545709 PMC3113731

[B36] Goodnight C. J. (1973). The Use of Aquatic Macroinvertebrates as Indicators of Stream Pollution. Trans. Am. Microscop. Soc. 92, 1–13. 10.2307/3225166 4735832

[B37] Günther J. (1976). Impulse Conduction in the Myelinated Giant Fibers of the Earthworm. Structure and Function of the Dorsal Nodes in the Median Giant Fiber. J. Comp. Neurol. 168, 505–531. 10.1002/cne.901680405 939820

[B38] Gustafsson D. R. Price D. A. Erséus C. (2009). Genetic Variation in the Popular Lab Worm *Lumbriculus variegatus* (Annelida: Clitellata: Lumbriculidae) Reveals Cryptic Speciation. Mol. Phylogenet. Evol. 51, 182–189. 10.1016/j.ympev.2008.12.016 19141324

[B39] Halfmann K. Crisp K. (2011). A Kinematic Study of Pulsation in the Dorsal Blood Vessel of the Blackworm, *Lumbriculus variegatus* . IMPULSE, 1–11. 21847432

[B40] Hamada Y. Bando T. Nakamura T. Ishimaru Y. Mito T. Noji S. (2015). Regenerated Leg Segment Patterns Are Regulated Epigenetically by Histone H3K27 Methylation in the Cricket *Gryllus bimaculatus* . Development 142, 2916–2927. 10.1242/dev.122598 26253405

[B41] Herlant-Meewis H. (1964). Regeneration in Annelids. Adv. Morphogen. 4, 155–215. 10.1016/b978-1-4831-9951-1.50008-5 5331921

[B42] Hessling R. Westheide W. (1999). CLSM Analysis of Development and Structure of the central Nervous System of *Enchytraeus crypticus* ("Oligochaeta", Enchytraeidae). Zoomorphology 119, 37–47. 10.1007/s004350050079

[B43] Holmquist C. (1976). Lumbriculids (Oligochaeta) of Northern Alaska and Northwestern Canada. Zool. Jahrb. Abt. Syst. Geog. Biol. Tiere 103, 377–431.

[B44] Holstein T. W. Hobmayer E. Technau U. (2003). Cnidarians: An Evolutionarily Conserved Model System for Regeneration? Dev. Dyn. 226, 257–267. 10.1002/dvdy.10227 12557204

[B45] Hornig E. C. (1980). Use of Aquatic Oligochaete, “*Lumbriculus variegatus*”, for Effluent Biomonitoring. United States Environmental Protection Agency. Industrial Environmental Research Laboratory. Available at: https://cfpub.epa.gov/si/si_public_record_Report.cfm?Lab=NERL&dirEntryID=43514 (Accessed June 28, 2021).

[B46] Hyman L. H. (1940). Aspects of Regeneration in Annelids. The Am. Naturalist 74, 513–527. 10.1086/280919

[B47] Isossimow V. V. (1926). Zur Anatomie des Nervensystems der Lumbriculiden. Zool. Jb. (Anat.) 48, 365–404.

[B48] Iwanow P. (1903). Die Regeneration von Rumpf- und Kopfsegmenten bei *Lumbriculus variegatus* Gr. Z. für wissenschaftliche Zoologie 75, 327–390.

[B49] Jamieson B. G. M. (1981). The Ultrastructure of the Oligochaeta. London, United Kingdom: Academic Press.

[B50] Killian M. D. Baker D. M. (2013). “Corralling Wiggling Worms—Collecting Data for a Multi-Week Laboratory on the Effect of Various Treatments on the Pulsation Rate of the Dorsal Vessel of California Blackworms (*Lumbriculus variegatus*),” in Proceedings of the 34th Conference of the Association for Biology Laboratory Education (ABLE), June 19–22, 2012. Editor McMahon K. (Chapel Hill: University of North Carolina), 499327–499335. Available at: http://www.ableweb.org/volumes/vol-34/?art=29 .

[B51] Knowles L. (2017). The Evolution of Myelin: Theories and Application to Human Disease. J. Evol. Med. 5, 1–23. 10.4303/jem/235996

[B52] Kostyuchenko R. P. Kozin V. V. (2021). Comparative Aspects of Annelid Regeneration: Towards Understanding the Mechanisms of Regeneration. Genes 12, 1148. 10.3390/genes12081148 34440322 PMC8392629

[B53] Kostyuchenko R. P. Kozin V. V. (2020). Morphallaxis versus Epimorphosis? Cellular and Molecular Aspects of Regeneration and Asexual Reproduction in Annelids. Biol. Bull. Russ. Acad. Sci. 47, 237–246. 10.1134/s1062359020030048

[B54] Kozin V. V. Kostyuchenko R. P. (2015). Vasa, PL10, and Piwi Gene Expression during Caudal Regeneration of the Polychaete Annelid *Alitta virens* . Dev. Genes Evol. 225, 129–138. 10.1007/s00427-015-0496-1 25772273

[B55] Krecker F. H. (1910). Some Phenomena of Regeneration in Limnodrilus and Related Forms. Z. Wissenschlaftliche Zoologie 95, 383–450.

[B56] Kumar A. Brockes J. P. (2012). Nerve Dependence in Tissue, Organ, and Appendage Regeneration. Trends Neurosci. 35, 691–699. 10.1016/j.tins.2012.08.003 22989534

[B57] LaRocca-Stravalle Z. Kauffman J. Gillen K. (2020). Poster Abstracts. Integr. Comp. Biol. 60, e269–e454. 10.1093/icb/icaa007

[B58] Lesiuk N. M. Drewes C. D. (1999). Autotomy Reflex in a Freshwater Oligochaete, *Lumbriculus variegatus* (Clitellata: Lumbriculidae). Hydrobiologia 406, 253–261. 10.1007/978-94-011-4207-6_25

[B59] Lesiuk N. M. Drewes C. D. (2001). Behavioral Plasticity and central Regeneration of Locomotor Reflexes in the Freshwater Oligochaete, *Lumbriculus variegatus*. I: Transection Studies. Invertebrate Biol. 120, 248–258. 10.1111/j.1744-7410.2001.tb00035.x

[B60] Lybrand Z. R. Martinez‐Acosta V. G. Zoran M. J. (2020). Coupled Sensory Interneurons Mediate Escape Neural Circuit Processing in an Aquatic Annelid Worm, *Lumbriculus variegatus* . J. Comp. Neurol. 528, 468–480. 10.1002/cne.24769 31502251

[B61] Lybrand Z. R. Zoran M. J. (2012). Rapid Neural Circuit Switching Mediated by Synaptic Plasticity during Neural Morphallactic Regeneration. Devel Neurobio 72, 1256–1266. 10.1002/dneu.20993 22021133

[B62] Martinez V. G. (2005). Cellular and Molecular Correlates of Neural Morphallaxis in *Lumbriculus variegatus* . Available at: http://hdl.handle.net/1969.1/3982 .

[B63] Martinez V. G. Manson J. M. B. Zoran M. J. (2008). Effects of Nerve Injury and Segmental Regeneration on the Cellular Correlates of Neural Morphallaxis. J. Exp. Zool. 310B, 520–533. 10.1002/jez.b.21224 PMC2754161 18561185

[B64] Martinez V. G. Menger G. J. III Zoran M. J. (2005). Regeneration and Asexual Reproduction Share Common Molecular Changes: Upregulation of a Neural Glycoepitope during Morphallaxis in *Lumbriculus* . Mech. Develop. 122, 721–732. 10.1016/j.mod.2004.12.003 15817228

[B65] Martinez V. G. Reddy P. K. Zoran M. J. (2006). Asexual Reproduction and Segmental Regeneration, but Not Morphallaxis, Are Inhibited by Boric Acid in *Lumbriculus variegatus* (Annelida: Clitellata: Lumbriculidae). Hydrobiologia 564, 73–86. 10.1007/s10750-005-1709-9

[B66] Martinez-Acosta V. G. Zoran M. J. (2015). “Evolutionary Aspects of Annelid Regeneration,” in eLS (Chichester: John Wiley & Sons). 10.1002/9780470015902.a0022103.pub2

[B67] Morgan T. H. (1901). Regeneration. Norwood, MA: Macmillan.

[B68] Morgulis S. (1909). Contributions to the Physiology of Regeneration. Archiv für Entwicklungsmechanik der Organismen 28, 396–439. 10.1007/BF02287014

[B69] Morgulis S. (1907). Observations and Experiments on Regeneration in *Lumbriculus* . J. Exp. Zool. 4, 549–574. 10.1002/jez.1400040405

[B70] Müller C. (1908). Regenerationsversuche an *Lumbriculus variegatus* und *Tubifex rivulorum* . Archiv für Entwicklungsmechanik der Organismen 26, 209–277. 10.1007/BF02162937

[B71] Müller O. F. (1774). Vermivm terrestrium et fluviatilium, seu animalium infusoriorum, helminthicorum et testaceorum, non marinorum, succincta historia. Havniæ: apud Heineck et Faber. Available at: https://www.biodiversitylibrary.org/item/50344 .

[B72] Mulloney B. (1970). Structure of the Giant Fibers of Earthworms. Science 168, 994–996. 10.1126/science.168.3934.994 5441033

[B73] Nikrad J. Tweeten K. (2014). Poster Abstracts. Integr. Comp. Biol. 54, e235–e375. 10.1093/icb/icu009

[B74] Niwa N. Akimoto-Kato A. Sakuma M. Kuraku S. Hayashi S. (2013). Homeogenetic Inductive Mechanism of Segmentation in Polychaete Tail Regeneration. Develop. Biol. 381, 460–470. 10.1016/j.ydbio.2013.04.010 23608458

[B75] Özpolat B. D. Bely A. E. (2016). Developmental and Molecular Biology of Annelid Regeneration: a Comparative Review of Recent Studies. Curr. Opin. Genet. Develop. 40, 144–153. 10.1016/j.gde.2016.07.010 27505269

[B76] Özpolat B. D. Sloane E. S. Zattara E. E. Bely A. E. (2016). Plasticity and Regeneration of Gonads in the Annelid *Pristina leidyi* . EvoDevo 7, 22. 10.1186/s13227-016-0059-1 27708756 PMC5051023

[B77] Phillips A. J. Dornburg A. Zapfe K. L. Anderson F. E. James S. W. Erséus C. (2019). Phylogenomic Analysis of a Putative Missing Link Sparks Reinterpretation of Leech Evolution. Genome Biol. Evol. 11, 3082–3093. 10.1093/gbe/evz120 31214691 PMC6598468

[B78] Phipps G. L. Ankley G. T. Benoit D. A. Mattson V. R. (1993). Use of the Aquatic Oligochaete *Lumbriculus variegatus* for Assessing the Toxicity and Bioaccumulation of Sediment-Associated Contaminants. Environ. Toxicol. Chem. 12, 269–279. 10.1897/1552-8618(1993)12[269:uotaol]2.0.co;2

[B79] Planques A. Kerner P. Ferry L. Grunau C. Gazave E. Vervoort M. (2021). DNA Methylation Atlas and Machinery in the Developing and Regenerating Annelid *Platynereis dumerilii* . BMC Biol. 19, 148. 10.1186/s12915-021-01074-5 34340707 PMC8330077

[B80] Purschke G. (2015). “Annelida: Basal Groups and Pleistoannelida,” in Structure and Evolution of Invertebrate Nervous Systems. Editors Schmidt-Rhaesa A. Harzsch S. Purschke G. (Oxford: Oxford University Press), 254–312. 10.1093/acprof:oso/9780199682201.003.0024

[B81] Quesada P. R. Miranda R. A. Arjona-Soberon J. Martinez-Acosta V. G. (2015). Development of a QPCR Assay to Evaluate Gene Transcripts Encoding Proteins Involved in *Lumbriculus variegatus* Regeneration. Integr. Comp. Biol. 55, e211–e356. 10.1093/icb/icv012

[B82] Randolph H. (1891). The Regeneration of the Tail in *Lumbriculus* . Zoologischer Anzeiger 14, 154–156.

[B83] Randolph H. (1892). The Regeneration of the Tail in *Lumbriculus* . J. Morphol. 7, 317–344. 10.1002/jmor.1050070304

[B84] Ribeiro R. P. Bleidorn C. Aguado M. T. (2018). Regeneration Mechanisms in Syllidae (Annelida). Regeneration 5, 26–42. 10.1002/reg2.98 29721325 PMC5911452

[B85] Roots B. I. Lane N. J. (1983). Myelinating Glia of Earthworm Giant Axons: Thermally Induced Intramembranous Changes. Tissue Cell 15, 695–709. 10.1016/0040-8166(83)90044-7 6648952

[B86] Rouhana L. Tasaki J. (2016). Epigenetics and Shared Molecular Processes in the Regeneration of Complex Structures. Stem Cell Int. 2016, 9. 10.1155/2016/6947395 PMC4670690 26681954

[B87] Ryan C. H. Elwess N. L. (2017). Graduate Student Commentary. AA 38, 38–43. 10.5195/aa.2017.185

[B88] Sardo A. M. Soares A. M. V. M. Gerhardt A. (2007). Behavior, Growth, and Reproduction of *Lumbriculus variegatus* (Oligochaetae) in Different Sediment Types. Hum. Ecol. Risk Assess. Int. J. 13, 519–526. 10.1080/10807030701341043

[B89] Sayles L. P. (1927). Origin of the Mesoderm and Behaviour of the Nucleolus in Regeneration in *Lumbriculus* . Biol. Bull. 52, 278–3121. 10.2307/1537098

[B90] Singh Patel S. Zunjarrao S. Pillai B. (2020). Neev, a Novel Long Non-coding RNA, Is Expressed in Chaetoblasts during Regeneration of *Eisenia fetida* . J. Exp. Biol. 223, jeb216754. 10.1242/jeb.216754 32098889

[B91] Smith F. (1905). Notes on Species of North American Oligochaeta. INHS Bull. 7 (1-10), 45–50. 10.21900/j.inhs.v7.404

[B92] Stephan-Dubois F. (1956). Migration and Differentiation of Neoblasts in Anterior Regeneration of *Lumbriculus variegatus* (Annelida, Oligochaeta). C R. Seances Soc. Biol. Fil 150, 1239–1242. 13383948

[B93] Stephenson J. (1930). The Oligochaeta. Oxford: Oxford University Press.

[B94] Stephenson J. (1924). XIV.-On Some Scottish Oligochæta, with a Note on Encystment in a Common Freshwater Oligochæte, *Lumbriculus variegatus* (Müll.). Trans. R. Soc. Edinb. 53, 277–295. 10.1017/S0080456800004026

[B95] Straus K. Chudler E. (2015). Botanical Heart Throbs: Heart Rate in Blackworms. Sci. Scope 039, 26–31. 10.2505/4/ss15_039_01_26

[B96] Takeo M. Yoshida-Noro C. Tochinai S. (2008). Morphallactic Regeneration as Revealed by Region-specific Gene Expression in the Digestive Tract of *Enchytraeus japonensis* (Oligochaeta, Annelida). Dev. Dyn. 237, 1284–1294. 10.1002/dvdy.21518 18393309

[B97] Tellez-Garcia A. A. Álvarez-Martínez R. López-Martínez J. M. Arellano-Carbajal F. (2021). Transcriptome Analysis during Early Regeneration of *Lumbriculus variegatus* . Gene Rep. 23, 101050. 10.1016/j.genrep.2021.101050

[B98] Turner C. D. (1935). The Effects of X-Rays on Anterior Regeneration in *Lumbriculus inconstans* . J. Exp. Zool. 71, 53–81. 10.1002/jez.1400710104

[B99] Turner C. D. (1934). The Effects of X-Rays on Posterior Regeneration in *Lumbriculus inconstans* . J. Exp. Zool. 68, 95–119. 10.1002/jez.1400680104

[B100] Tweeten K. A. Anderson A. (2008). Analysis of Cell Proliferation and Migration during Regeneration in *Lumbriculus variegatus* (Clitellata: Lumbriculidae). BIOS 79, 183–190. 10.1893/0005-3155-79.4.183

[B101] Tweeten K. Abitz A. (2012). Patterns of Cleavage and Gastrulation in Embryos of Freshwater Oligochaetes from the *Lumbriculus* Complex. Integr. Comp. Biol. 52, e202–e356. 10.1093/icb/icr007

[B102] Tweeten K. A. Morris S. J. (2016). Flow Cytometry Analysis of DNA Ploidy Levels and Protein Profiles Distinguish between Populations of *Lumbriculus* (Annelida: Clitellata). Invertebr Biol. 135, 385–399. 10.1111/ivb.12150

[B103] Tweeten K. A. Reiner A. (2012). Characterization of Serine Proteases of *Lumbriculus variegatus* and Their Role in Regeneration. Invertebr. Biol. 131, 322–332. 10.1111/ivb.12002

[B104] Tweeten K. Myers M. Norton C. Gildensoph L. Phillips M. J. Wygal D. (2007). “Animating a Biology Curricuulm with Research,” in Developing and Sustaining a Research-Supportive Curriculum: A Compendium of Successful Practices. Editors Karukstis K. K. Elgren T. E. (Washington, DC: Council on Undergraduate Research), 122–127.

[B105] Tweeten K. Vang C. (2011). Poster Abstracts. Integr. Comp. Biol. 51, e158–e269. 10.1093/icb/icr007

[B107] von Haffner K. (1928). Über die Regeneration der vordersten Segmente von *Lumbriculus* und ihre Fähigkeit, ein Hinterende zu regenerieren. Z. für wissenschaftliche Zoologie 132, 37–72.

[B106] von Haffner K. (1931). Die überzähligen Bildungen des Körperstammes von *Lumbriculus variegatus* Müll. und ihre kausale Analyse. W. Roux' Archiv F. Entwicklungsmechanik 123, 649–681. 10.1007/BF01380649 28354039

[B108] von Wagner F. (1906). Beitrage zur Kenntnis der Regenerationsprozesse bei *Lumbriculus variegatus*. II. Teil. Zool. Jb. Abt. Anat. U. Ontog. 136, 255–318.

[B109] von Wagner F. (1900). Beitrage zur Kenntnis der Regenerationsprozesse bei *Lumbriculus variegatus*. I. Teil. Zool. Jb. Abt. Anat. U. Ontog. 22, 41–156.

[B110] von Wagner F. (1897). Zwei Worte zur Kenntnis der Regeneration des Vorderdarmes bei *Lumbriculus* . Zoologischer Anzeiger 20, 69–70.

[B111] Walther J. B. Walther J. B. (1971). Funktionelle anatomie der dorsalen riesenfaser-systeme von *Lumbriculus terrestris* L. (Annelida, Oligoehaeta). Z. Morph. Tiere 70, 253–280. 10.1007/BF00302028

[B112] Wenzel O. (1923). Beiträge zur Kenntnis der normalen und regenerativen Cytologie des *Lumbriculus variegatus* Gr. Lotos - Z. fuer Naturwissenschaften 71, 243–267.

[B113] Williams E. B. Mann K. G. (1993). “Peptide Chloromethyl Ketones as Labeling Reagents,” in Methods in Enzymology Proteolytic Enzymes in Coagulation, Fibrinolysis, and Complement Activation Part A: Mammalian Blood Coagulation Factors and Inhibitors (Cambridge, MA: Academic Press), 503–513. 10.1016/0076-6879(93)22031-A 8412812

[B114] Zattara E. E. (2020). “Axial Regeneration in Segmented Animals: A Post-Embryonic Reboot of the Segmentation Process,” in Cellular Processes in Segmentation Evolutionary Cell Biology (Boca Raton, FL: CRC Press), 255–292. 10.1201/9780429423604-13

[B126] Zattara E. E. Bely A. E. (2011). Evolution of a Novel Developmental Trajectory: Fission is Distinct From Regeneration in the Annelid *Pristina leidyi* . Evol. Develop. 13, 80–95. 10.1111/j.1525-142X.2010.00458.x 21210945

[B115] Zattara E. E. Bely A. E. (2015). Fine Taxonomic Sampling of Nervous Systems within Naididae (Annelida: Clitellata) Reveals Evolutionary Lability and Revised Homologies of Annelid Neural Components. Front. Zool 12, 8. 10.1186/s12983-015-0100-6 25960761 PMC4424535

[B116] Zattara E. E. Bely A. E. (2016). Phylogenetic Distribution of Regeneration and Asexual Reproduction in Annelida: Regeneration Is Ancestral and Fission Evolves in Regenerative Clades. Invertebr Biol. 135, 400–414. 10.1111/ivb.12151

[B117] Zattara E. E. Özpolat B. D. (2021). “Quantifying Cell Proliferation during Regeneration of Aquatic Worms,” in Developmental Biology of the Sea Urchin and Other Marine Invertebrates: Methods and Protocols Methods in Molecular Biology. Editors Carroll D. J. Stricker S. A. (New York, NY: Springer US), 163–180. 10.1007/978-1-0716-0974-3_10 33074540

[B118] Zattara E. E. (2012). Regeneration, Fission and the Evolution of Developmental Novelty in Naid Annelids. PhD thesis. College Park: University of Maryland, 197. 10.13140/2.1.2054.4967

[B119] Zattara E. E. Turlington K. W. Bely A. E. (2016). Long-term Time-Lapse Live Imaging Reveals Extensive Cell Migration during Annelid Regeneration. BMC Dev. Biol. 16, 6. 10.1186/s12861-016-0104-2 27006129 PMC4804569

[B120] Zhinkin L. (1932). Die Regeneration bei *Lumbriculus variegatus* nach Einwirkung von Röntgenstrahlen. Zoologischer Anzeiger 100, 34–43.

[B121] Zhinkin L. (1935). Über den determinierenden Einfluss des Nervensystems auf die Regeneration bei *Lumbriculus variegatus* . Arch. Russes d’Anatomie, d’Histologie d’Embryologie 14, 715–719.

[B122] Zhinkin L. (1936). Über die abhängige Differenzierung des Nervensystems während der Regeneration bei *Lumbriculus variegatus* . W. Roux' Archiv F. Entwicklungsmechanik 134, 251–261. 10.1007/BF00573982 28354439

[B123] Zoran M. J. Martinez V. G. (2009). “ *Lumbriculus variegatus* and the Need for Speed: A Model System for Studies of Rapid Escape, Regeneration and Asexual Reproduction,” in Annelids as Models Systems in the Biological Sciences. Editor Shain D. (Hoboken, NJ: Wiley-Blackwell Publishers), 185–204.

[B124] Zoran M. J. Drewes C. D. Fourtner C. R. Siegel A. J. (1988). The Lateral Giant Fibers of the Tubificid worm, *Branchiura sowerbyi*: Structural and Functional Asymmetry in a Paired Interneuronal System. J. Comp. Neurol. 275, 76–86. 10.1002/cne.902750107 3170791

[B125] Zoran M. J. Drewes C. D. (1987). Rapid Escape Reflexes in Aquatic Oligochaetes: Variations in Design and Function of Evolutionarily Conserved Giant Fiber Systems. J. Comp. Physiol. 161, 729–738. 10.1007/BF00605014

